# Indicators of Horse Welfare: State-of-the-Art

**DOI:** 10.3390/ani10020294

**Published:** 2020-02-13

**Authors:** Clémence Lesimple

**Affiliations:** Univ Rennes, Normandie Univ, CNRS, EthoS (Éthologie Animale et Humaine)—UMR 6552, F-35380 Paimpont, France; lesimple.c@gmail.com; Tel.: +33-223-237-766

**Keywords:** animal welfare, horse, indicators

## Abstract

**Simple Summary:**

Scientific interest in animal welfare was initially driven by popular emotional, ethics and political concerns. It has now become an important societal question and all the stakeholders agree on the necessity to rely on unambiguous scientific evidence for evaluation and making decisions. Animal welfare is defined as a chronic state reflecting an animal’s subjective perception of its situation indicated by behavioural, postural and physiological parameters. Because of their multiple statuses (as farm, leisure, sport or pet animals), horses experience a large variety of more or less adequate environmental conditions that present risks of impairing their welfare. The aim of this review is to disentangle welfare parameters and to differentiate reliable animal-based indicators of horses’ welfare from potential signals of acute sickness, discomfort, temporary states of pain, stress or emotion that are not based on popular beliefs in order to provide the equine industry with appropriate guidelines and recommendations.

**Abstract:**

Animal welfare is defined as a chronic state reflecting an individual’s subjective perception of its situation. Because it is possible to be in a good welfare state and nevertheless experience acute fear or pain, and conversely, short-term positive emotions can be experienced during impaired welfare states, welfare as a chronic state has to be clearly distinguished from temporary states related to emotions, pain or stress. The evaluation of non-verbal individuals’ welfare state, particularly in interspecific situations, is a real challenge that necessarily implies animal-based measures and requires multidisciplinary scientifically validated measures. In the last decade, studies investigating horses’ welfare flourished together with new measures that were not always scientifically tested before being used. At a time were legal decisions are made on animal welfare, it is crucial to rely on reliable welfare indicators in order to prevent false evaluation. The aim of this review is to identify the scientifically tested and reliable indicators of horses’ welfare (e.g., body lesions, apathy, aggressiveness, stereotypic behaviours) from signals of temporary states related to acute pain emotions or stress and from popular beliefs, in order to give the scientific community and the horse industry accurate evaluation tools.

## 1. Introduction

### 1.1. From Animal Protection to Animal Welfare

The first major measure of animal protection legislation appeared in the 1800s in the United Kingdom, when parliament ratified the Cruel Treatment of Cattle Act [1] and made it an offence, punishable by fines up to five pounds or two months imprisonment, to “beat, abuse, or ill-treat any horse, mare, gelding, mule, ass, ox, cow, heifer, steer, sheep or other cattle”. Similarly, the United States [2] and France [3] included the offense of cruelty to animals (including, but not only, killing, wounding and maiming) in their laws. However, only in 1976 did the French law recognize that animals are sentient beings that should be kept under conditions fitting their biological needs [4]. However, despite a century of amendments and law modifications, change was slow, as the notions of cruelty, abuse and ill-treatment are vague and highly subjective. 

The current concept of “animal welfare” emerged from popular emotional reactions elicited by Ruth Harrison’s book [5], which prompted the British government to create a commission to investigate the breeding conditions of intensively farmed animals. Under the direction of Professor F.W.R. Brambell, the commission gathered information about the practices inherent to intensive animal production in the UK, by visiting consenting facilities and collecting the oral/written testimonies of various publics (15 days). This inquiry resulted in a report [6] aiming at “examining the conditions in which livestock are kept under systems of intensive husbandry and to advise whether standards ought to be set in the interests of their welfare, and if so what they should be” in the UK. Their conclusions were limited, according to the authors, in domain (intensive breeding only, excluding non-intensive breeding, transport and slaughtering conditions or privately kept animals), geographic area (Great Britain only, excluding Northern Ireland) and time (“what appears tolerable today may come to be considered intolerable in the future”) [6]. It stated that animals should at least be free to stand up, lie down, turn around, stretch limbs and interact with conspecifics. In 1979, the Farm Animal Welfare Council (FAWC, UK) used this report as a basis to institute the concept of the Five Freedoms principle to regulate farm animals’ welfare, while “encouraging alternative systems of livestock husbandry which are ethically acceptable to the concerned public and economically competitive with the existing system of intensive production” [7]. Both the Brambell report and the Five Freedoms principle were based more on observations of environmental conditions (mainly spatial organization) than on the animal itself and were presented not as scientific guarantees of preserved welfare, but as minimal requirements without which the animals’ welfare is necessarily impaired [6,7,8]. The Five Freedoms concept hence stems from emotional awareness, political urgency and ethical issues rather than from real scientific concerns about animal welfare and have been updated in order to meet popular and ethical expectations [8]. Interestingly, even though the European Community agrees on the necessity of scientific-based evidence concerning animal welfare, its current legislation in this domain is still based on the Five Freedoms [9].

### 1.2. Scientific Approaches of Animal Welfare

Unlike most scientific areas that emerged from the interest and curiosity of pioneer scientists, research on animal welfare emerged from emotional and ethical societal concerns (e.g., [9,10]). In the dictionary, welfare has been defined as a “chronic pleasant state resulting from both body needs’ satisfaction and calmness of the mind” [11]. Although satisfaction of body needs can be assessed relatively easily, evaluation of the “calmness of the mind” of non-verbal individuals is more challenging. The scientific community has developed several concepts to characterize and appreciate animal welfare. Behavioural approaches state that animals should live accordingly to their nature [12] and should be able to perform (or at least not prevented in the long term from performing) all necessary behaviours without aversion or deprivation (e.g., [13]). However, perceptions of what a “necessary behaviour” is differ and some authors consider it as the possibility to express the animal’s entire natural behavioural repertoire (e.g., [14]), while other authors consider it as the ability to perform what an animal “desires” (subjective feelings) or needs (biological needs) without supplementary effort (e.g., [15]). Since animals have been recognized as sentient beings, the presence/absence of emotions has been included in animal welfare considerations. Thus, in order to ensure good welfare, animals should be protected from having negative emotions (e.g., fear, pain), and should be able to experience positive emotions [16,17].

Physiological approaches based on the concepts of homeostasis and allostasis have been developed. Homeostasis is defined as an approximately constant state which varies only within tolerable limits [18], whereas allostasis is defined as “stability through changes” [19]. The homeostasis model suggests that an animal’s welfare can be insured only by protecting it from environmental changes (e.g., [18]), but the allostasis principle is based on an organism’s constant internal and external changes aiming to maintain a homeostatic state. In both cases, welfare is ensured when the organism is in a long-term stable state, including (allostasis) or excluding (homeostasis) necessary variations. These different approaches emphasize the chronic nature of animal welfare as well as its complexity and multi-facetted essence.

### 1.3. Welfare, Emotions and Pain

As stated above, the term welfare refers to the persistent state of an individual over time (i.e., chronic) and can be either positive or negative according to its subjective perception of its environment. On the contrary, emotions (positive or negative) or pain (unpleasant sensation felt in some part of the body, [11]) are mainly related to acute and temporary events, hence restricted in time (except for chronic pains that are mainly considered as disabilities [20]). Feeling pain or negative emotions (e.g., fear) on a regular basis over time can lead to a negative welfare state, whilst a succession of positive emotions is most certainly a necessary precursor of a positive welfare state. However, an individual in a negative welfare state can experience positive emotions, and conversely, an individual in a positive welfare state can experience negative emotions. Thus, living under naturalistic conditions does not imply that an animal is free from constraints (e.g., thermal, feeding). However, experiencing too hot/cold temperatures or lack of food, as long as these deprivations are within the animals’ adaptation possibilities, induces only occasional or temporary perturbations (positive or negative) rather than welfare impairment [9]. When investigating welfare conditions, especially those of non-verbal individuals, it is crucial to adopt a chronic perspective and to evaluate the long-lasting situation of the organism rather than its temporary state, to prevent drawing unfounded conclusions. 

Whatever the approach adopted, authors commonly accept that welfare is a complex multifaceted concept reflecting an organism’s subjective perception of a chronic situation.

## 2. Notion of Welfare Indicator

Assessing the welfare of a non-verbal individual, in particular, in an interspecific context, is a real challenge and requires the identification of reliable visible indicators reflecting an individual’s subjective perception of its chronic situation. An indicator can be defined as “a thing that indicates the state or level of something” [11]. A potential animal welfare indicator must include the following principles: validity (i.e., be meaningful for animal welfare and measure what it is supposed to), reliability (i.e., produce consistent results when used by different observers) and feasibility (i.e., easy to use in the field) [21,22]. Thus, before being considered as true welfare indicators, potential candidates have to be clearly related to an individual’s actual welfare state and have to be proven to be consistent and reliable in the field.

In order to respect the “validity” principle (it measures what it is meant to), welfare indicators have to yield information about an individual’s subjective perception of its situation, and consequently, have to be based on the animal itself rather than on its environment. Indeed, description of an animal’s environment can only indicate risks of welfare impairment, not the actual welfare state of an animal [23,24] and thus, cannot be considered as a welfare indicator. According to Veasey [25], it is mandatory, when working on animal welfare, to be able to refer to a standard population, living in optimal conditions and presenting a positive welfare state, in order to make appropriate comparisons. Finally, one must keep in mind that the welfare state is a subjective perception, leading to potential differences between two conspecifics in the same situation; if the many expressions of welfare improvement/impairment are not considered, the choice of indicators might lead to extensive discrepancies between evaluations of the same situation (e.g., [26]). To evaluate an animal’s welfare state accurately, it is thus essential to rely on multiple scientifically validated indicators, covering all the facets of welfare (behavioural, psychological and physiological) and reflecting an individual’s chronic state (e.g., [27]). These indicators have to be based on the characteristics and needs of the species (welfare indicators are not necessarily interchangeable between species) and reflect the animal’s perception (free from human anthropomorphic considerations) [13]. Finally, when evaluating the welfare of a group of animals (an entire facility), the proportion of individuals presenting welfare impairment signals must be taken into account (see Hausberger et al, same issue).

As they are considered as livestock, work, sport, leisure and pet animals, horses are submitted to a large array of more or less adapted environmental conditions (inducing more or less important impacts on their welfare). Consequently, to improve horses’ welfare, reliable welfare indicators reflecting horses’ chronic states must be identified and differentiated from potential indicators of temporary states, such as pain, emotion or acute stress. The aim here is to review the signals currently used to assess horse welfare, to identify reliable indicators, and consequently, to provide the equine industry with consistent welfare assessment tools. Thus, if all the signals and indicators included in the current welfare evaluation protocols as well as the ones currently used in the literature about horses’ welfare are evaluated here, the relevance of these protocols is not at stake here and will not be discussed in this review (see Hausberger et al., same issue). 

The term “indicators” is used only for reliable scientifically established measures (scientifically related to other welfare impairment signs and providing consistent results between observers) concerning horses’ welfare. The term “signal” is used for other measures (related to acute states, ambiguous or vague descriptions). As mentioned above, as the only way to evaluate an individual’s welfare state is to consider the individual itself, environmental measures will not be taken into consideration here. We focus first on health-related, physiological, postural and behavioural welfare indicators, and then present signals potentially related to welfare states, but that require further investigation. Finally, we discuss parameters used for welfare assessment based on subjective evaluations and popular beliefs that are only vaguely described.

## 3. Indicators of Horses’ Welfare 

This part focuses on reliable indicators of horses’ welfare, i.e., the signals revealing horses’ chronic state that have been formerly scientifically related to other signals and have been proven as consistent between observations (Table 1).

### 3.1. Health-Related Indicators 

Physical injury is considered as one of the primary causes of welfare impairment, as it affects the individual in its integrity. First of all, the presence of **work-related body lesions** or marks of former lesions (naked or white hair areas) located at places of friction with working equipment (e.g., harness, bit, bridle, saddle), which reflects chronic use of poor-fitting equipment or work overload (e.g., [28]), is related to poor body condition scores, apathy and systemic health abnormalities including diarrhoea and dehydration [28,29,30], revealing welfare impairment. Using a classical 3-point scale (superficial: hair loss, broken skin, deep/swelling wounds, e.g., [28,30,62,63,64]), Burn et al. [65] found moderate to excellent agreement between six observers concerning the presence and severity of work-related body lesions on 80 horses. Therefore, the presence of work-related body lesions indicates an impaired welfare.

With the worldwide expansion of overweight horses [31,66] and the poor ability of owners to assess their horses’ condition correctly [32,66], it is important to have a practical tool to evaluate their body condition. The **Body Condition Score** (BCS) is based on visual evaluation of body fat by palpation of various areas of the animal’s body in order to evaluate its “physical state”, ranging from extreme thinness to extreme obesity. Two different scales are currently used, the first in 5 points (1–5, [67]) and the second in 9 points (1–8, e.g., [33]). The 9-point scale is used more frequently and is positively correlated with horses’ body-fat percentage [32,33], and blood variables (including leptin, insulin, glucose and triglyceride rates). Equines with high BCS (7 < X < 8, considered obese) are more prone to suffer physiological disorders (e.g., hyperinsulinemia [31], insulin resistance: [34]). Recent research highlighted differences between BCS and body weight ranging from 2.2% to 4.9% of the horse’s total body weight (3 to 39 kg) according to the breed [66]. However, the aim of BCS is to evaluate a horse’s body condition rather than its actual weight, with both evaluations reflecting different factors. Although BSC has proven to be repeatable when used by experienced assessors [68], its scoring is less reliable when used by non-experienced assessors, in particular, for the higher scores [32]. BCS has proven to assess horses’ body condition reliably and thus potential welfare impairments when used by experienced observers, but requires adequate suitable training to ensure its efficiency.

Elementary visual examination of a horse’s body can alert an observer to potential welfare deterioration when work-related lesions are present and BC scores are extreme (low or high). 

### 3.2. Postural Indicators

Body postures are long used to assess animals’ acute emotional and chronic states [69]. In some cases, chronic health disorders are related to **typical postures**, such as laminitis with an arched body that takes the weight off the fore feet [20]. **Ear positions** are good indicators: if ears pointing backwards are associated with negative states, including pain (e.g., [20] for a review) or agonistic social interactions [70], they are also associated with chronically restricted conditions—the time spent with ears backwards in homogenous conditions (i.e., foraging) is related to chronically restricted living conditions (strict social isolation [35]) and chronic welfare impairment (e.g., vertebral disorders, stereotypic behaviours [36,37]), while horses under naturalistic conditions are almost never observed with such ear positions. Although time spent with ears backwards while foraging is not related to one particular context, it is a reliable indication of impaired welfare. Finally, a recent study showed that the prevalence of vertebral disorders evaluated by sEMG (see below) was related to a particular **neck shape** [38]: markers were placed along the spine in a sagittal plane, and located at musculoskeletal cues, enabling consistent reproduction of positioning, and horses were photographed when standing and walking in hand. The evaluations of the horses’ neck postures were based on the measurement of the angles between the markers. The values of these angles were significantly correlated to the prevalence of vertebral disorders: the hollower the neck, the more the spine was affected. On the contrary, horses with a rounded neck had no vertebral disorders [38,39]. 

Here again, visual examination of a horse’s body yielded reliable information concerning that horse’s welfare state and potential injuries.

### 3.3. Physiological Indicators

Changes in welfare state, either in a positive or in a negative way, can affect the physiology of an individual, but physiological signals of welfare are difficult to apprehend in a chronic perspective as: (1) it can take a long time before a modification of the environment is reflected by physiological parameters (i.e., blood count), it is difficult to know for sure what environmental conditions are effective, and (2) short-term internal changes (e.g., hormonal) can reflect chronic as well as acute situations. However, the presence of chronic vertebral disorders, related to lameness [71,72,73], changes in gait [71,72] or asymmetry of the pelvic bony prominences [72,74], is commonly considered as one of the most widespread causes of impairment of working horses’ welfare [39,72,74,75,76] and can induce **muscular hypertonicity** along the spine that can be assessed by manual palpation [36,38,40,76,77]) or static electromyography (sEMG) [38]. Evaluations of back pain/vertebral disorders presented a strong agreement between practitioners regardless of their occupational background (e.g., [38,40,77]), as well as between methods [38]. Although muscular activity cannot be estimated by simple visual examination, its evaluation constitutes a reliable indicator of horses’ welfare [39].

### 3.4. Behavioural Indicators

Sub-optimal living conditions are known to have an impact on animals’ behaviour, including modification of their time-budget (i.e., time spent in different activities) and on their behavioural repertoire (apparition or loss of behaviours). 

#### 3.4.1. Modifications of Horses’ Behavioural Repertoire 

One of the most common and recognized behavioural indicators of welfare impairment is the presence of **stereotypic or abnormal repetitive behaviours** (SB/ARB). SB/ARB are invariant repetitive behavioural sequences, performed with no obvious goal or function [41], that appear under sub-optimal living conditions [41,42]. The development of horses’ SB/ARB has been widely investigated and, although genetic aspects are most certainly involved (e.g., [43]), these behaviours only emerge in inappropriate environments. Management during a horse’s early development may increase (or on the contrary prevent) risks of SB/ARB. Thus, early weaning and social isolation facilitate SB/ARB, while late weaning and group housing (especially in the presence of adults) have protective effects [78]. SB/ARB can appear as soon as 3 weeks after stabling [44]. Inappropriate management of adult horses has also been shown to facilitate SB/ARB emergence (social isolation [44,45]; spatial restriction [43], visual horizon [46,47], feeding [48,49], working conditions [50,51]); whereas efforts to provide conditions closer to natural conditions [47,79] or to decrease potential frustration [47,48] decreased their prevalence. Expression of SB/ARB is related to physiological and health disorders [46,54,80,81]. Following the coping hypothesis, SB/ARB would be a way for horses (and other animals) to tolerate their environment better (e.g., [82]). Despite the fact that having to “cope” with the environment means that the environment is inappropriate, the results concerning the coping efficiency of SB/ARB are not clear: although the acute stress state of stereotypic horses seemed to worsen when they were prevented from stereotyping [83], cortisol concentrations of stereotypic and non-stereotypic horses did not differ significantly [84,85,86,87,88], suggesting that stereotypic horses do not cope better with sub-optimal environments than their non-stereotypic counterparts. Evaluation of the presence and prevalence of SB/ARB was consistent over time [89]; therefore, reliably indicating impaired welfare of horses [35,42,90]. However, although some ARB are well known (e.g., weaving, cribbing) and their recordings present good inter-observer agreement [62], other ARB are more subtle and are more difficult to identify [91]. Many studies of horses focus on “traditionally” recognized stereotypic behaviours (i.e., cribbing, windsucking, weaving, pacing, pawing, head tossing/nodding), but it is crucial to take into account other more subtle ARB (e.g., compulsive licking/biting/kicking, other head movements, tongue movements, complex sequences, see also [35,42,91]) to ensure a reliable evaluation. The expression of SB/ARB by stereotypic horses seems to be strongly context-dependent (in individual stalls rather than in pasture, with a large outside visual horizon rather than in indoor stalls, mostly before meals [42,46,47]), highlighting the importance of choosing the right time to assess their prevalence.

When under sub-optimal conditions, horses can develop an **apathetic state**, related to depressed-like syndromes [28,29,52,65,92], characterized by sensorial withdrawal (visual and tactile [28,92]) and a general flattened posture [92]. This syndrome, first identified for horses working in developing countries [28,30], has also been identified in riding school horses in France [35,52,92], and is related to the occurrence of health (work-related wounds [29,30], low BCS and lameness [28]), physiological (depressed cortisol levels [92]) and behavioural/psychological (anhedonia: [52]) disorders. However, Burn et al. [65] found only a moderate inter-observer agreement for their horse population. An apathetic state is a reliable indicator of impaired welfare but it requires suitable training for assessors to identify it consistently.

#### 3.4.2. Modification of the Horses’ Time Budget

Observations of feral horse populations enabled the construction of the natural “time-budget” of this species, i.e., the percentage of time devoted to diverse activities [70]. Modifications of this time-budget occur in inappropriate environments and may reveal welfare impairment. Under natural conditions, horses spend most of their time grazing/foraging (67%–75% of their time), resting (including sleeping: 15%–25%), observing their environment (6%–10%) or moving (8%), the remaining time being occupied by maintenance activities, social interactions and reproduction [70]. When horses are placed under inappropriate living conditions, this time budget may be modified. Thus, horses living under inappropriate environmental conditions were observed to decrease time **resting** (including sleep) [47,55,81], increase their **aggressiveness** towards conspecifics [53,55,92] and increase their **active locomotion** patterns (including active walk, trot, canter, [53,55,56]). Lack of rest/sleep in situations favouring the expression of stereotypic behaviours [47] was related to an increase of pathological behaviours [59], whereas horses with deprived social competencies increased their aggressiveness and their reproduction abilities decreased [57,93].

#### 3.4.3. Other Behavioural Indicators

Behavioural **reactions towards humans**, either in human–horse relation tests [58] or during daily interactions (e.g., [28,29,30]) also yield information concerning the horses’ welfare. Horses living under constraining environmental conditions can become aggressive towards humans [44,60] and this has been related to higher rates of vertebral disorders [36]. On the contrary, horses were less aggressive towards the experimenter when their back was healthy [36]. Finally, a **cognitive bias** was shown to reflect horses’ welfare state. Thus, a recent study [37] showed that horses in a poor welfare state (evaluation based on the expression of SB/ARB, ear position, aggressive reactions to humans and vertebral disorders) were also more pessimistic when in the presence of an ambiguous stimulus, whereas horses in a good welfare state were clearly optimistic. 

Observations of a horse’s behaviour either in its usual situations or during behavioural experiments testing particular reactions offer a reliable insight of its current welfare state. A frequent criticism of behavioural observation is the risk of an observer-related bias, which indeed exists, but is minimized when the observers have been trained correctly, the behavioural patterns are described precisely, inter-observer reliability is checked and the methods used are rigorous and validated [61].

### 3.5. Conclusions

Thus, reliable indicators of horses’ welfare exist, with most of them being detectable by visual examination of a horse’s body or by observation of their behaviour. However, in order to prevent mistaken or false evaluations, and consequently, detrimental management decisions, the observers have to be adequately trained to identify and to recognize real welfare alterations.

## 4. Signals Potentially Related to Welfare State 

As stated above, when evaluating the welfare state of an animal, its chronic (long-term) state must be taken into consideration and the reliability of an evaluation must be guaranteed (do we measure what we are supposed to?). The signals discussed below can be related to both chronic and acute contexts and may be included in the scope of welfare evaluation if coupled with reliable indicators, but their reliability for assessing welfare on their own is not ensured (Table 2).

### 4.1. Health-Related Signals 

**Lameness**, potentially related to acute pain or discomfort [94], can reveal chronic problems [73,75] and thus become a welfare issue. Clinical evaluation of lameness is generally based on visual scoring. However, the scales and methodologies used vary greatly between studies, involving different detection scales including evaluation points that are more or less well described [124,125]. Apart from overt lameness (i.e., when the horse is unable to use its leg or has a clear unbalanced neck movement), inter-observer agreement varied from poor [124,125] to good [126] between reports depending on the evaluation method, on the speed, on the type of movement (straight or in a circle) and on the experience of the observers. In addition to being potentially related to acute pain or discomfort and not chronic welfare, the presence of lameness seems to be difficult to assess reliably. Given its non-specificity and its rather complicated evaluation, its presence signals a potential problem, but cannot be considered a welfare indicator per-se. **Prolapses** (uterine, anal or vaginal) are included in most welfare evaluation protocols and considered as welfare impairment signals. Little scientific literature on this subject exists, most of it reporting case studies (e.g., [95]). The presence of prolapses whatever the type, reveals a severe violation of the individual’s health and rather than welfare impairment, constitutes a clear veterinary emergency.

In the same way, the evaluation of **hoof condition** is often included in welfare evaluation protocols, together with the presence of “medical” symptoms such as **coughing** or **discharges** (e.g., ocular, nasal, genital), all likely to occur in both acute and chronic contexts, and can even not relate to any impact on the animal’s welfare. Inappropriate hoof condition is likely to increase the incidence of foot and limb problems as well as lameness and may be related to unsuitable housing, feeding or laminitis episodes (e.g., [96] for a review). However, the evaluation of hoof condition has to be used cautiously: the horses’ hoof wall is particularly well-adapted to its functional role and has internal properties leading to natural fractures when walking on hard soils, thus inducing an uneven aspect of the hooves that does not reflect any welfare impairment [97]. On the contrary, inadequate trimming or shoeing may be visually imperceptible but alter a horse’s integrity and induce musculoskeletal injuries [127]. Moreover, most studies were based on clinical assessment methods that cannot be used practically in the field [128]. Except for extreme visible cracks, long wall or overt lameness, it is difficult to determine whether the condition of a horse’s hooves reveals its welfare state. Fluid discharges and coughing can be related to either acute (e.g., [98]) or chronic (e.g., [99]) disorders. Easy to assess, their presence cannot be considered a reliable welfare indicator, as they might reflect temporary discomfort. However, in the case of high prevalence inside a stable or continuous expression by one individual, coughing and fluid discharges point to potential veterinary issues.

Finally, the prevalence of gastric ulceration is very important in domestic horses [129,130]. As it is difficult to assess, it remains often untreated, leading to chronic abdominal pain and subsequent welfare impairment. However, apart from behavioural modifications (see Section 3.4.1), gastric ulcerations assessment requires performing invasive or expansive examination of the gastro-intestinal tract, and no clear indicators exist. 

Broadly speaking, if the presence of wounds (other than work-related), marks of former lesions and overt signals of pain, pathology or health-related issues, in general, may be related to temporary states, it has still to alert on potential welfare issues. As such, it should elicit a thorough welfare inspection of the animal, using reliable welfare indicators.

### 4.2. Postural Signals

Amongst the signals used more and more to assess horses’ welfare emerge the evaluation of facial expressions. Used for a long time to identify humans’ emotional states, evaluation of facial expression is based on the description of facial movements due to underlying musculature [100]. Previous reports showed that an animal’s face is not necessarily the best place to look when assessing its emotional state and could even lead to misleading results [101]. Attempts have been made to characterize horses’ facial expressions when in acute pain either induced [102], following veterinarian interventions [63] or at work [108]. In addition, tests have been made to use horses’ facial expressions to evaluate their welfare [62]. Although the accuracy of facial expressions to assess horses’ pain is not clear (see [20]), its relevance in case of chronic states (welfare) has never yet been tested: the different patterns used in the horse facial expression scale were never scientifically related to actual welfare impairment signals and further studies are clearly needed.

### 4.3. Physiological Signals

Hormonal assays have been developed to investigate horses’ welfare. In particular, **cortisol concentrations** (the so called “stress hormone”) are commonly used, through blood, faecal or more recently, hair sampling. Plasma cortisol levels, currently used to investigate the effect of acute stressors because of its quick release peak after stimulation, are used to evaluate chronic situations. In most cases, plasma cortisol levels of horses presenting potential welfare impairments (chronic health disorders: e.g., [103]; chronic stress: e.g., [44]; depressed-like horses [92]) are low. However, no differences were found between the cortisol levels of stereotypic and non-stereotypic horses [85,86,87,88] or stereotyping and non-stereotyping phases [87], but the cortisol levels of horses with chronic laminitis were higher than those of controls [104]. In addition to these contradictory results, blood plasma analyses necessitate sampling by venipuncture that could have an impact on cortisol levels. More recently, non-invasive cortisol assays by faecal, saliva and hair sampling have been developed for horses, avoiding any potential sampling effects, but they yielded contradictory results. Pawluski et al. [103] found that horses with impaired welfare had lower faecal cortisol levels (e.g., vertebral disorders, ears backwards, abnormal cell counts), but no differences were found between horses presenting SB/ARB [84] or gastric ulceration [105] and their healthy counterparts, or between socially isolated and group-housed horses [78]. Basal salivary cortisol concentrations of stereotypic horses were lower [106] or were similar [85] to those of non-stereotypic horses. Moreover, Harewood [107] showed that although social isolation could elicit strong behavioural reactions, salivary cortisol levels did not increase. Results concerning hair cortisol may be biased due to temporal changes in hair growth as well as possible effects of local cortisol production in the hair follicles [131], and may change with the hair and according to its body location [132]. Whatever the sampling methodology, results concerning cortisol assays in relation to the assessment of horses’ welfare are contradictory, and although cortisol levels likely reflect temporary unpleasant states, they do not seem reliable to evaluate welfare. Recent analyses of **serotonin and oxytocin levels** related to positive (oxytocin increase [133]; serotonin increase [109]) or negative (serotonin decrease [110]) emotional states were developed for assessing horse welfare. However, results are contradictory: some studies report lower basal serotonin levels for horses with chronic health disorders than for healthy horses (chronic diseases, laminitis [104]) and lower for cribber than for non-cribber horses [87]. On the other hand, no differences were found between cribbing and non-cribbing phases for cribber horses [87], or between stereotypic and non-stereotypic horses (all SB/ARB included [88]). In other species, hormonal profiles were shown to vary according to a wide range of factors, including, but not only, genetic [111,112] and gender [112,134] effects, which could explain such discrepancies. Here again, hormonal levels do not seem to inform reliably about horses’ welfare and appear to be related more to intermittent states.

Haematological parameters, and in particular, **white cell counts,** are affected by chronic diseases (ex: chronic laminitis [113]) and by prolonged stressful conditions (transportation [118]). A recent study of over 2000 horses showed that horses’ welfare was negatively correlated with the neutrophil/lymphocytes (N/L) ratio [114]: horses with lower welfare scores presented higher N/L ratios. However, the horses’ welfare score used in this study included both animal-based indicators and environmental observations (reflecting potential and not actual welfare alterations). Further investigations including only animal-based scores are necessary to confirm that the N/L ratio is a reliable indicator of welfare impairment.

Finally, attempts have been made to use **heart rate** (HR) and **heart rate variability** (HRV), first developed for the assessment of emotional situations (e.g., [135]), to evaluate potential welfare alterations. However, although HR and HRV vary in emotionally challenging situations, the results regarding welfare assessment were more controversial. Thus, HR and HRV of stereotypic horses were either lower than [106] or did not differ from those of [86] their non-stereotypic counterparts; and Lebelt et al. [87] found that cribber horses’ HR decreased during cribbing periods. On the other hand, social and spatial restriction did not influence HR [107].

Scientific evidence shows thus that hormonal and heart rate scores can reflect potentially intermittent unpleasant states but they cannot be used reliably to evaluate welfare. Abnormal blood cell counts seem to be an interesting lead, but the results for the N/L ratio have to be confirmed.

### 4.4. Behavioural Signals

Some behaviours may be expressed in positive, negative and more ambiguous situations, and for this reason, have to be considered carefully together with their context of emission, and cannot necessarily be considered as welfare indicators. **Yawning**, often considered as a relaxation signal and used as a positive welfare expression, can be expressed in contexts of motor relaxation, but may also be triggered by stress and negative contexts [136]. Increased rates of yawning by horses were found following the administration of soothing products leading to muscular relaxation [137], but also in ambiguous or frustrating situations [115] or chronic diseases (e.g., [116]) and have even been related to the occurrence of SB/ARB [138]. In the same way, **play** has long been considered as an indicator of positive welfare [117], but increasing evidence shows a potential relationship with inappropriate living conditions (see [139] for review). Young horses express play exclusively [70] under favourable conditions (e.g., [119]), but play can also be triggered by social isolation [140]. Adult horses in a wild and feral population are hardly ever seen playing [70], while domestic horses are regularly reported to play. Also, locomotor rebound happens in animals under spatial restriction when they have access to larger spaces [56,141], and this is regularly considered as play. Thus, the presence of play, although potentially related to immediate short-term positive emotions, also raises questions concerning potential chronic restrictions in a horse’s life and hence, its welfare. Reports recently related horses’ **attentional state** to the prevalence of vertebral disorders. Calm observation of their environment, reflecting a positive attentional state [70], was less frequent by horses presenting vertebral disorders than by controls [120], and horses with impaired welfare were more unresponsive to their environment [28,30]. Although horses with impaired welfare seem to neglect their environment, over-attention—including vigilance—often associated with increased locomotion, is not at stake here. **Vacuum chewing**, sometimes defined as a relaxation signal in horses, is considered a displacement behaviour when in stressful situations for other species [121] and appears in contexts frustrating (e.g., [122,136]) for horses. All these behaviours are likely to reflect both liberation from chronic constraints and potential positive temporary states. As a consequence, they cannot be considered as welfare indicators, but their occurrence, in particular, when repeated over time can indicate welfare impairment. 

### 4.5. Acoustic Signals

Horses’ acoustic communication has not been thoroughly investigated, and although whinnies are known to encode both social [123] and emotional information [142], information carried by non-vocal communication (snore, snort, blows) are less clear. Because descriptions are imprecise, snorts and snores have been confused for a long time, both were considered as indicative of high emotional states [143,144]. Recent reports seem to show that **snorts** are expressed more in positive contexts [145,146]. Additionally, the rates of snort production decreased significantly when the horses’ welfare state was impaired [146]. The production of snorts could hence inform about the valence of a horse’s perception of its environment.

### 4.6. Conclusions

Most of the signals described in the section above are not specific of welfare impairment and can indicate acute and chronic states. Thus, their presence cannot be considered as signals of welfare impairment but should lead to further examination of the animal. Clear identification of the methodologies and context of observation must be provided.

## 5. Signals Lacking Scientific Validation 

The use of signals that are scientifically validated, based on popular beliefs and not sufficiently described to be reliably identified can lead to inappropriate evaluations and mistaken conclusions, and are thus not helpful. The signals described below, although they might be related at some point to welfare impairment, have not yet been sufficiently characterized and require additional scientific testing before being used.

### 5.1. Health-Related Elements

The fact that animal welfare concerns are related to emotional and ethical matters [9,10] means that substantial risks related to subjectivity and tradition are likely to become involved when evaluating welfare, and this is at the expense of scientific-based evidence, thus potentially leading to incorrect conclusions. The signals described in this section might be related to welfare impairment or discomfort, but are mostly based on feelings and lack precise description and/or scientific validation. 

Because they are external signals that can be recognized by rapid visual examination, several health-related signals are commonly used to evaluate horses’ welfare, even if they have not been described precisely or shown to be reliably related to any chronic situation. The presence of swollen joints, abnormal breathing or excessive sweating or shivering for example, are often considered as signals of impaired welfare. However, these elements are only vaguely described (“exaggerate effort to breathe”, “profuse sweating”, [62]) or only defined in comparison to the other limb for the swollen joint. Moreover, rather than signals of chronic welfare impairment, these elements seem more informative about acute pathological states. In the same vein, the assessment guidelines concerning hair coat condition, also described as a signal of potentially impaired welfare, are imprecise (“local alteration”, “changing coat” [62]) and have not previously been related to any physiological, behavioural or health-related welfare-impairment indicator. 

### 5.2. Behavioural and Postural Elements

Elements used by humans to describe a horse’s behaviour or attitude (annoyed, at ease, happy, undesirable behaviours) as well as vaguely described, and acute state-related behavioural patterns (tail swishing, fear behaviours, pain behaviours) are sometimes included in the evaluation of a horse’s welfare [62,63]. Such vague evaluations, based on an assessor’s subjective perception of an animal’s state, never related to welfare impairment indicators, are likely to lead to incorrect conclusions. 

### 5.3. Physiological Elements

When an individual is in acute stress, a process of sympathetically mediated vasoconstriction induces an almost immediate drop of its skin and body temperatures [147]. Thus, these last few years, animals’ body temperature, measured in particular at the level of the eyes, has been used increasingly to assess the emotional valence and intensity of acute events (see [148] for a review). Increase of horses’ eye temperature is related to other physiological measures of acute stress (blood and saliva cortisol levels [149]). The impact of acute environmental conditions [150], fear [151] and pain (tighten noseband: e.g., [152]) on eye temperature has been investigated. However, although links between acute stress and skin/body temperature seem clear [149] and can be explained physiologically [147], currently there is no evidence of an impact of a chronic (positive or negative) situation. Thus, body temperature should be considered as an indicator of acute modification but not as an indicator of welfare. 

### 5.4. Conclusions

Although some of these elements may reflect inadequate management of horses and consequently, welfare impairment, the lack of scientific precision and validation makes them difficult to be used reliably. In order to ensure reliable evaluation and to prevent incorrect conclusions/decisions, references to subjective human perceptions, poorly described elements or popular beliefs must be avoided. Further research has to ensure the validity and reliability of these elements before using them as welfare indicators.

## 6. General Conclusions

Animal welfare opens a very interesting but a very difficult research area, where strong emotional and popular beliefs [9,10] risk overriding scientific evidence. Horses’ particular status elicits strong emotional reactions and although scientists and legislators recognize the necessity to rely on scientific evidence, old habits and popular beliefs die hard. The main aim of all the research on horses’ welfare is to ensure that every decision will lead to an improvement of their living conditions. Taking this into consideration, and to prevent making premature decisions that could be harmful, it is crucial to conform to scientific standards and to the definition of welfare.

Thus, when investigating horses’ welfare, one should ensure that (1) the signals recorded truly reflect a chronic state; (2) the signals are meaningful for animal welfare and measure what they are supposed to; (3) the definition and description of the signals are clear and can be identified beyond question [21,22]; (4) that the signals are scientifically validated and related to other welfare indicators. An animal can experience temporary pain or fear although its welfare is good and, on the contrary, it can experience a positive mood temporarily although its welfare is impaired. The assessment of fear, pain or emotional level has to be clearly differentiated from welfare evaluation. Reliable and visible indicators of horses’ welfare do exist, and these should be exclusively used when evaluating horses’ welfare. Of course, additional less specific signals could be included in welfare evaluation, and could alert to potential impairment, but further research is needed in order to ensure their reliability. Finally, subjective human perception should clearly be removed from welfare evaluations, as it introduces emotional biases with a high risk of drawing incorrect conclusions and making harmful decisions. Before presenting or publicizing novel indicators of horse welfare, their reliability and validity must be more effectively checked [21,22] so as to avoid making incorrect evaluations and incorrect decisions [26,102].

## Figures and Tables

**Table 1:** Indicators of horses’ welfare. X: specifies whether the indicator may reveal a temporary state or a veterinary issue as well as welfare. (X): indicates whether signal may be a predictor of chronic welfare state, temporary state or a veterinary issue when combined with other signals. References are given in italics for each signal.

Type of Signal			Welfare State	Temporary State
**Health Related**		Body lesion [28,29,30]	x	x
		Body Condition Score [31,32,33,34]	x	x
		Specific postures [20]	x	x
**Postural**		Ear position [35,36,37]	x	x
		Neck shape [38,39]	x	(x)
**Physiological**		Muscular hypertonicity [39,40,41]	x	x
**Behavioural**	Behavioural repertoire	Stereotypic/Abnormal Repetitive Behaviours (SB/ARB) [42,43,44,45,46,47,48,49,50,51]	x	x
		Apathy [28,29,30,52,53]	x	(x)
	Time-budget	Rest [48,54,55]	x	x
		Aggressiveness [52,55,56,57,58]	x	x
		Active locomotion [55,56,59]	x	
	Other	Reaction to humans [28,29,30,36,45,60,61]	x	x
		Cognitive bias [37]	x	x

**Table 2:** Signals potentially related to horses’ welfare. X: indicates whether signal reveals a chronic welfare state, temporary state or a veterinary issue. (X): indicates whether signal may be a predictor of chronic welfare state, temporary state or a veterinary issue when combined to other signals. References are given in italics for each signal.

Type of Signal		Chronic Welfare State	Temporary State
**Health-Related**	Lameness [73,75,94]	(x)	x
	Prolapse [95]		x
	Hoof condition [96,97]	(x)	x
	Cough/Discharges [98,99]	(x)	
**Postural**	Facial expressions [20,62,63,100,101]		x
**Physiological**	Cortisol (faecal, blood, hair, saliva) [44,78,84,85,86,87,88,102,103,104,105,106,107]	(x)	x
	Serotonin/oxytocin [87,88,108]		
	White cell count [109,110,111]	(x)	x
	HR/HRV [86,87,104,105,112]		x
**Behavioural**	Yawning [113,114]	(x)	x
	Play [70,115,116]	(x)	x
	Attentional state [28,30,70,117]	(x)	x
	Vacuum chewing [118,119]	(x)	x
**Acoustic**	Snort [120,121,122,123]	(x)	x
